# Control of Mechanical Properties of Thermoplastic Polyurethane Elastomers by Restriction of Crystallization of Soft Segment

**DOI:** 10.3390/ma3125097

**Published:** 2010-12-01

**Authors:** Ken Kojio, Mutsuhisa Furukawa, Yoshiteru Nonaka, Sadaharu Nakamura

**Affiliations:** 1 Materials Science and Engineering Department, Faculty of Engineering, Nagasaki University, 1-14 Bunkyo-machi, Nagasaki 852-8521, Japan; 2 Nagasaki University, 1-14 Bunkyo-machi, Nagasaki 852-8521, Japan; E-Mail: furukawa@nagasaki-u.ac.jp; 3 Materials and Science Department, Graduate School of Science and Technology, Nagasaki University, 1-14 Bunkyo-machi, Nagasaki 852-8521, Japan

**Keywords:** thermoplastic polyurethane elastomer, crystallization, microphase separation, mechanical properties

## Abstract

Mechanical properties of thermoplastic polyurethane elastomers based on either polyether or polycarbonate (PC)-glycols, 4,4’-dipheylmethane diisocyanate (1,1’-methylenebis(4-isocyanatobenzene)), 1,4-butanediol, were controlled by restriction of crystallization of polymer glycols. For the polyether glycol based-polyurethane elastomers (PUEs), poly(oxytetramethylene) glycol (PTMG), and PTMG incorporating dimethyl groups (PTG-X) and methyl side groups (PTG-L) were employed as a polymer glycol. For the PC-glycol, the randomly copolymerized PC-glycols with hexamethylene (C_6_) and tetramethylene (C_4_) units between carbonate groups with various composition ratios (C_4_/C_6_ = 0/100, 50/50, 70/30 and 90/10) were employed. The degree of microphase separation and mechanical properties of both the PUEs were investigated using differential scanning calorimetry, dynamic viscoelastic property measurements and tensile testing. Mechanical properties could be controlled by changing the molar ratio of two different monomer components.

## 1. Introduction

Of the various thermoplastic elastomers, polyurethane elastomers (PUE) are pivotally important, since they can give us quite wide-range physical properties. The PUE usually possesses a segmented structure, which consists of a soft segment formed with a polymer glycol and a hard segment formed with a diisocyanate and curing agent. Finally, the polyurethane molecules microphase-separate at a nanometer scale [1,2,3,4]. Both the microphase-separated structure and mechanical properties of the PUEs are influenced by a chemical structure and weight fraction of both components, a molecular weight, polydispersity of the soft segment component, thermal history, and so on [1,2,3,4,5,6,7,8,9,10,11,12,13,14,15,16,17,18,19,20,21,22,23].

For the PUEs, the soft segment can be classified into either an ether series or an ester, including carbonate ones. Generally, it is known that polyether glycol has a good resistance for hydrolysis and poor property for UV irradiation [11,12,13,14,15,16], and that polyester glycol has the opposite trends for these properties. The polycarbonate (PC)-glycol is a relatively new one bearing both features [17,18,19,20,21,22,23]. Thus, they are employed depending on the situation of the practical uses. However, there is an open question as to the control of the mechanical properties of various types of PUEs.

In this study, we synthesized thermoplastic polyurethane elastomers based on either polyether or PC-glycols, 4,4’-dipheylmethane diisocyanate (1,1’-methylenebis(4-isocyanatobenzene)), 1,4-butanediol. In order to control the mechanical properties, we incorporated monomers with side methyl and dimethyl groups for the polyether glycol and different numbers of methylene chain for the PC-glycol. Then, the influence of restriction of crystallization of polymer glycols on the degree of microphase separation and mechanical properties are discussed.

## 2. Experimental Section

### 2.1. Synthesis of Polyether Glycol-Based PUEs

Poly(oxytetramethylene) glycol (PTMG, *M*_n_ = *ca.* 2000) was used as a control polymer glycol. Two other kinds of polymer glycol with methyl and dimethyl groups (*M*_n_ = *ca.* 2000) were employed. One is copolymerized with tetrahydrofuran (THF) and neopentyl glycol, and the other one is done with THF and 3-methyl THF. They were denoted as PTG-X and PTG-L, respectively. PTG-X and PTG-L were kindly supplied from Asahi Kasei Co. Ltd. and Hodogaya Chemical Co. Ltd., respectively. In the synthetic process of PTG-X, THF was polymerized by a ring-opening reaction, and then neopentyl glycol was subsequently added. Thus, many dimethyl groups exist at the chain end region in the PTG-X molecules. Two kinds of PTG-X with methyl group contents of 10 and 20 mol % (PTG-X10 and PTG-X20) were employed. On the contrary, PTG-L was copolymerized by a ring-opening reaction of THF and 3-methyl THF. Hence, the side methyl groups exist randomly in the PTG-L molecules. Two kinds of PTG-L with methyl group contents of 8 and 20 mol % (PTG-L8 and PTG-L20) were employed. *M*_n_ is the number-average molecular weight and was determined by hydroxyl value.

The PUEs were synthesized from the polymer glycol, 4,4’-diphenylmethane diisocyanate (1,1’-methylenebis(4-isocyanatobenzene)) (MDI: Nippon Polyurethane Industry, Co., Ltd.), and 1,4-butanediol (BD: Wako Chemical, Co., Ltd.) by a prepolymer method. The polymer glycols were dried with dried nitrogen under a reduced pressure. Prepolymers were prepared from the polymer glycols and MDI with a ratio of *K* = [NCO]/[OH] = 3.3 at 80 °C for 3 hours under nitrogen atmosphere. The extent of reaction was pursued by a di-*n*-butylamine titration method. We took the method described in ASTM D 5155. After finishing the reaction, the prepolymer was placed in vacuum to remove the air inside. The prepolymer and a curing agent were mixed well with a ratio of [NCO]_pre_/[OH] = 1.05 for 90 s and a viscous product was poured into a mold constructed by a spacer of 2 mm thickness and two aluminum plates heated at 80 °C where [NCO]_pre_ is the number of isocyanate groups in the prepolymer. The PUEs were taken after 2 h curing, and were post-cured at 80 °C for 24 h in air.

### 2.2. Synthesis of PC-Glycol-Based PUEs

Randomly copolymerized PC-glycols (*M*_n_ = 2000, Asahi Kasei Chemicals, Co., Ltd.) were employed in this study. The nomenclature consists of 4 digits. The first two numbers indicate the number of methylene unit (C_4_ and C_6_). The third number relates the molar ratio of the C_4_ unit. The last number indicates a thousand digit of molecular weight of PC-glycols. The PUEs were prepared from polymer glycol, MDI and BD by a prepolymer method. The polymer glycols were dried with dried nitrogen gas under reduced pressure. Prepolymers were prepared by mixing polymer glycols and MDI with the ratio of *K* = [NCO]/[OH] = 2.05 and 3.05 at 80 °C for 5 minutes. Prepolymer and BD as a curing agent were mixed well with the ratio of NCO INDEX = [NCO]_pre_/[OH]_BD_ = 1.05 for 90 seconds, where [NCO]_pre_ is the number of isocyanate groups in the prepolymer. The viscous mixture was poured into the centrifugal mold and reacted at 110 °C for 1 hour. After removal from the centrifugal mold, the sample was post-cured at 100 °C for 24 hours.

### 2.3. Characterization of PUEs

Hardness was determined as the International Rubber Hardness Degree (IRHD) using a durometer with the A scale, which is used for rubbers in the normal hardness range. These data were measured in general accord with the procedures described in ISO 7619-1 and JIS K 6253.

Wide angle X-ray diffraction (WAXD) measurement was carried out for the PUEs at various strains to investigate the elongation-induced crystallization behavior during the elongation process. True strain was calculated using the magnitude of initial length and elongation by calipers. An X-ray source used was an MX18H (Mac Science, Co. Ltd., Japan) and voltage and current were set to be 40 kV and 200 mA. WAXD patterns were obtained with an imaging plate (DIP 2000, Mac Science, Co. Ltd., Japan). Exposure time was 300 s for each measurement. WAXD profiles were obtained by integral along the Bragg’s angle around equatorial line with 5° in the azimuthal direction.

Differential scanning calorimetric (DSC) measurement was performed to understand the thermal behavior of the PUEs. DSC thermograms were obtained with a DSC (DSC 8230, Rigaku Denki, Co., Ltd. Japan) in the temperature range from −140 to 250 °C with a heating rate of 10 °C min^−1^ under nitrogen atmosphere. As-prepared samples were simply cooled down around −140 °C, then measurement was started. *T*_g_ was taken at the temperature at which half the increase in baseline shift has occurred.

Tensile testing was performed with an Instron type tensile tester (AGS 100A, Autograph, Shimadzu, Japan) at 20 °C. The sample dimensions were 60.0 mm × 5.0 mm × 0.4 mm. The initial length and elongation rate were set to be 30 mm and 10 mm min^−1^, respectively. Since thin samples were employed, we could obtain exact strain, but tensile strength showed slightly smaller values than usual.

Temperature dependence of dynamic viscoelastic properties was measured with a dynamic mechanical analyzer (DMS 6100, Seiko Instruments, Co., Ltd.). The size of samples used is 30 mm × 5 mm × 2 mm. Measurement was performed in the temperature range from −150 to 250 °C with a heating rate of 2 °C min^−1^ with nitrogen atmosphere. The imposed strain and frequency were set to be 0.2% and 10 Hz, respectively.

## 3. Results and Discussion

### 3.1. Polyether Glycol-Based PUEs

**Figure 1:**
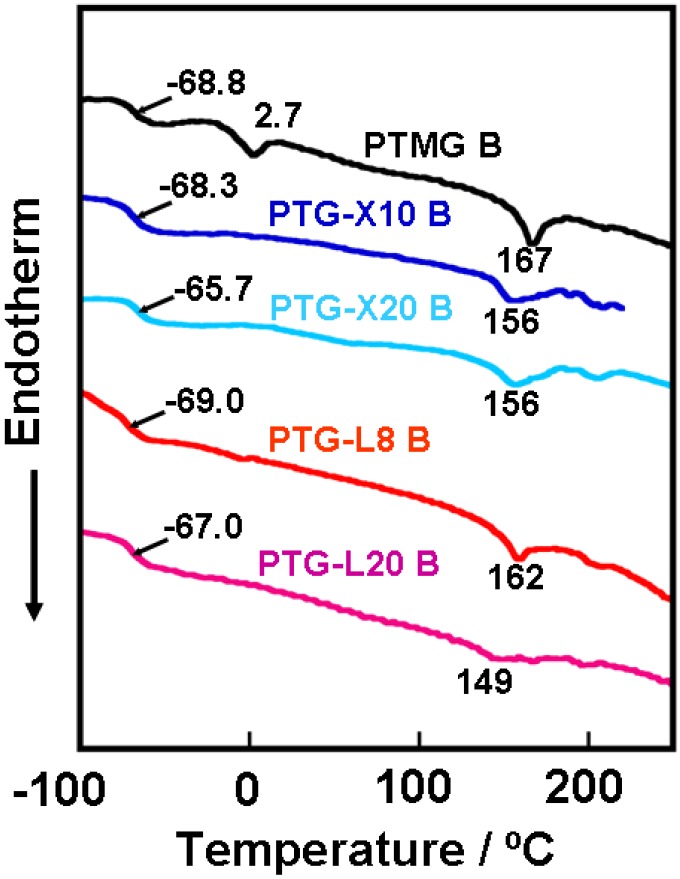
DSC curves for the PTMG, PTG-X and PTG-L based PUEs.

Nomenclature denotes the types of polymer glycol and curing agent used like PTMG B. Figure 1 shows the DSC curves for the PTMG, PTG-X and PTG-L based PUEs. Glass transition temperature (*T*_g_) of soft segment and melting point of hard segment crystals were observed around −65 and 150 °C for all PUEs, respectively. For PTMG B, one can see the obvious endothermic peak of the melting of soft segment at 2.7 °C. However, no clear peaks were observed for the other PUEs. These results clearly suggest that incorporation of the methyl and dimethyl groups into the polymer glycols are quite effective on the crystallizability of soft segment in the PUEs even under no elongation.

**Figure 2:**
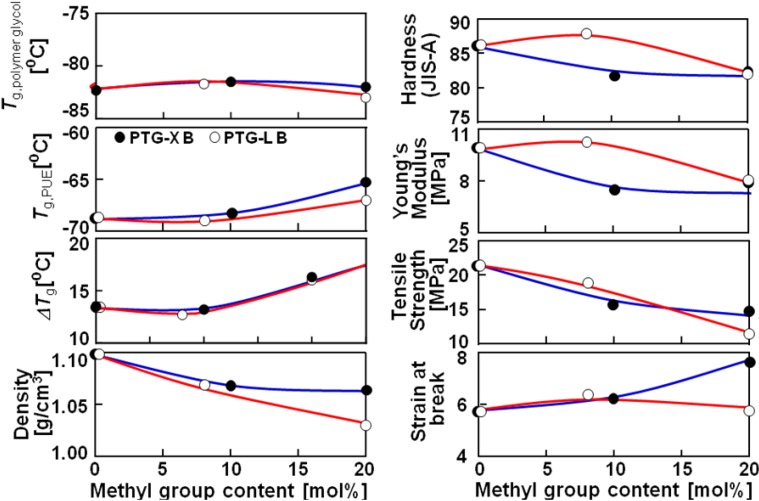
Methyl group content dependence of *T*_g_, density, hardness and mechanical properties of polymer glycol and PTMG, PTG-X and PTG-L-based PUE.

Figure 2 shows the methyl group content dependence of *T*_g_, density, hardness and mechanical properties of the PTMG, PTG-X and PTG-L based PUEs. Δ*T*_g_ was defined as a subtraction of *T*_g_ of the soft segment in the PUEs and that of original polymer glycols (*T*_g_,_polymer glycol_). Δ*T*_g_ increased with increasing methyl group content. It is well known that the increasing *T*_g_ of the soft segment in PUE systems means an increasing miscibility of the two components on account of the molecular interaction between the soft and hard segments. Furthermore, the melting temperature of the hard segment decreased with increasing methyl group content. Therefore, it is likely to consider that the phase separation of the PUEs became weaker with increasing methyl group content. The discussion on mechanical properties will be given in a later section.

**Figure 3:**
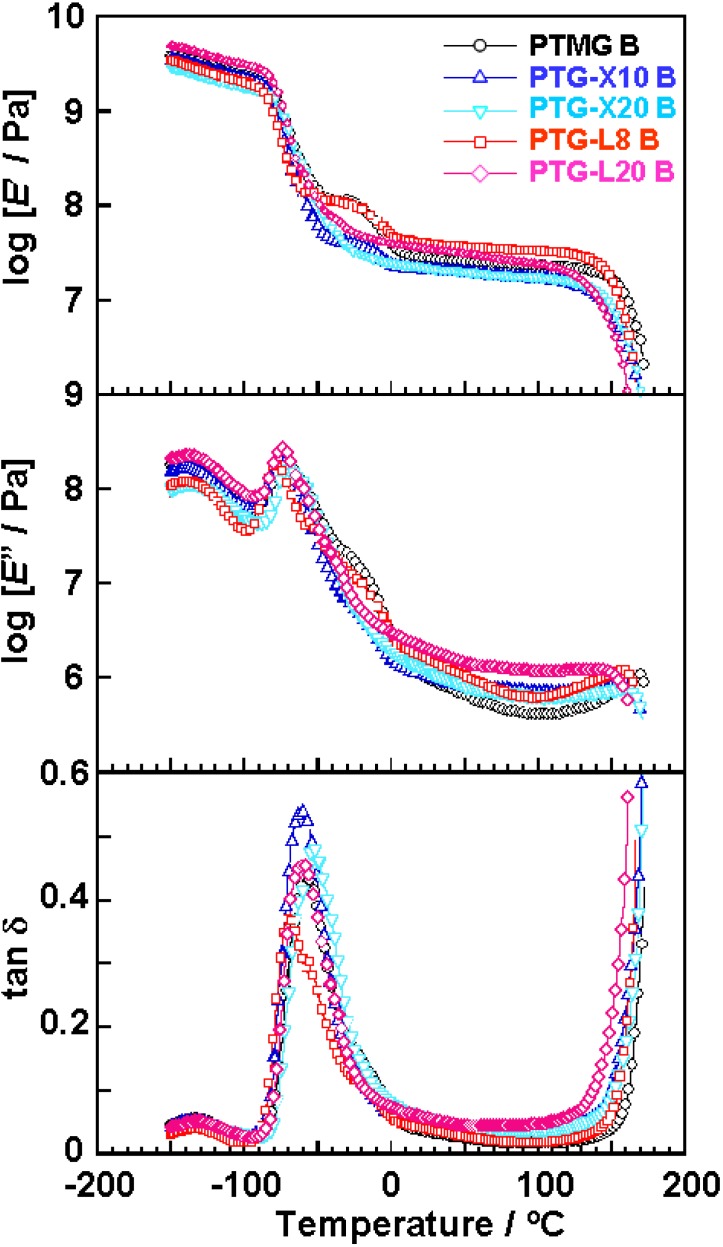
Temperature dependence of storage modulus (*E*’), loss modulus (*E*”) and loss tangent (tan *δ*) of PTMG, PTG-X and PTG-L-based PUEs measured at 10 Hz.

Figure 3 shows the temperature dependence of dynamic viscoelastic properties of the polyether glycol-based PUEs. The *E*’ decreased at around −80 °C because of α-relaxation of the soft segment. For PTMG B, PTG-X10 B and PTG-L8 B, a shoulder was clearly observed at around −20 °C. This is due to recrystallization of the soft segment during the heating process. Shoulders for PTG-X10 B and PTG-L8 B were smaller than for PTMG B. These results clearly indicate that incorporation of side methyl groups suppresses the recrystallization of the soft segment. On the other hand, since no shoulder was observed for PTG-X20 B and PTG-L20 B in this temperature region, the soft segment could not recrystallize during the heating process due to enough amounts of side methyl groups. All the PUEs exhibited a rubbery plateau region in the range from 0 to 150 °C. The terminal temperature of PTMG B was the highest of the five PUEs. This is because the hard segment chains in PTMG B formed well-ordered crystalline domains as clarified by DSC.

**Figure 4:**
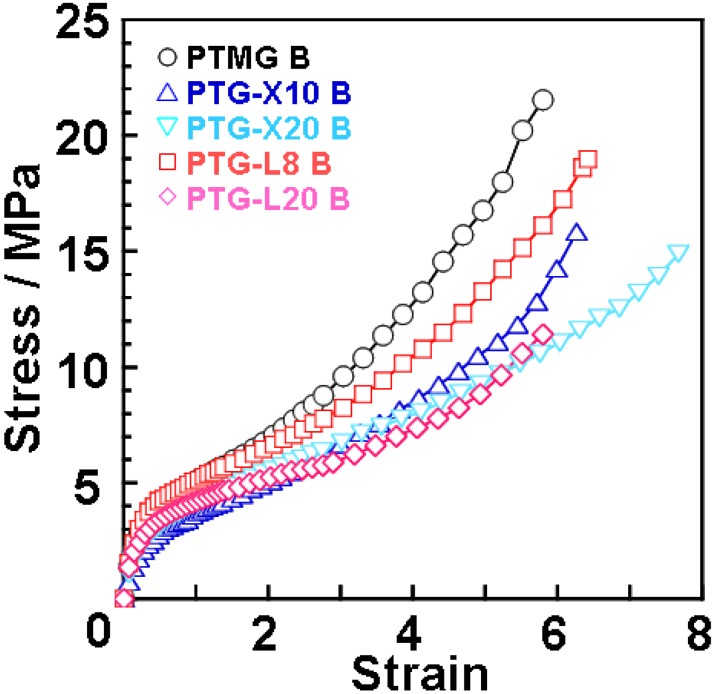
Stress-strain curves for PTMG, PTG-X, PTG-L-based PUEs measured at 20 °C.

Figure 4 shows stress-strain curves for the polyether glycol-based PUEs at 20 °C. The methyl group content dependence of Young’s modulus, tensile strength and strain at break are given in Figure 2. Young’s modulus decreased with increasing side methyl group content. This is because the incorporation of the side methyl group content causes restriction of crystallization of the soft segment chain and a decrease in physically crosslinking points due to a decrease in the degree of microphase separation. On the contrary, tensile strength and elongation at break decreased and increased, respectively. These changes can be attributed to the steric hindrance of dimethyl and methyl groups of the soft segment chains in the expanded state. That is, for PTMG B, PTG-X10 B and PTGL8 B, the soft segment chains can be crystallized well with increasing strain. In contrast, for PTG-X20 B and PTG-L20 B, dimethyl and methyl groups effectively disrupt crystallization of the soft segment and a phase mixing trend causes molecular slipping between the hard segments. Discussion about elongation-induced crystallization will be stated with the result of WAXD measurement. Stress-strain test revealed that introduction of methyl groups to polymer glycol is quite effective not only for the initial property (Young’s modulus) but also the elongated state (tensile strength and strain at break).

Dependence of methyl group content of the PTG-X series is slightly larger than that for the PTG-L ones. Dimethyl groups of the PTG-X preferentially exist near the end hydroxyl groups and are more bulky compared with methyl groups, resulting in a larger effect on molecular aggregation state of the hard segment. For this reason, the PTG-X series exhibited larger dependence on methyl group content in the case of no elongation.

**Figure 5:**
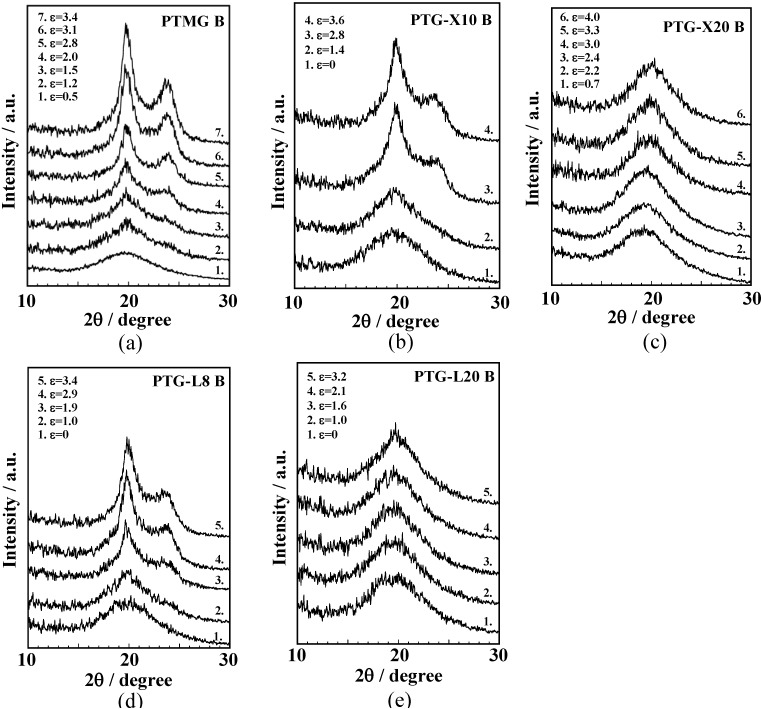
WAXD profiles of (**a**) PTMG B, (**b**) PTG-X10 B, (**c**) PTG-X20 B, (**d**) PTG-L8 B and (**e**) PTG-L20 B at various strains.

WAXD measurement was carried out at various strains to investigate the change in molecular aggregation structure of the PUEs during the elongation process. Figure 5 (a)–(e) shows the WAXD profiles for the polyether glycol-based PUEs at various strains. Amorphous halo was observed at 2θ = 20° for all the PUEs at the initial state. With increasing strain, crystalline peaks were observed at 2θ = 20° and 24° for PTMG B, PTG-X10 B and PTG-L8 B. The minimum strain, at which diffraction peaks were observed, was *ca.* 1.0. For PTMG B, PTG-X10 B and PTG-L8 B, two crystalline peaks were clearly observed in the higher strain region. Intensities of these crystalline peaks increased with increasing strain. It was reported that the crystal system of PTMG is monoclinic and its lattice constants are a = 0.559 nm, b = 0.890 nm, c = 1.207 nm, β = 134.2° [24]. Thus, PTMG shows (020) and (100) at 2θ = 19.8° and 24.2° around 20°. Therefore, it seems likely to consider that these two peaks observed for the PTMG B, PTG-L8 B and PTG-X10 B PUEs can be assigned to the crystal formed with the soft segment chain. Hence, it is conceivable that the elongation-induced crystallization occurred during the elongation process for PTMG B, PTG-X10 B and PTG-L8 B. In contrast, no crystalline peaks were observed for PTG-X20 B and PTG-L20 B even at a higher strain, although a peak position of the amorphous halo shifted to larger 2θ region. This indicates that the soft segment chain oriented toward the elongation direction and the molecular distance of the soft segment chain in the PTG-X20 B and PTG-L20 B PUEs became smaller, but they did not crystallize due to the strong steric hindrance of side methyl groups.

### 3.2. PC-Glycol-Based PUEs

We shall discuss the results on the PC-glycol-based PUEs. The nomenclature consists of four digits. The first two numbers indicate the number of methylene units (C_4_ and C_6_). The third number relates the molar ratio of the C_4_ unit. That is, 4,602, 4,652, 4,672 and 4,692 refer to 0, 50, 70, and 90 mol % C_4_ unit, respectively. The last number indicates the thousand digit of the molecular weight of poly(carbonate) glycols. To evaluate microphase separation in detail, two PUEs with different formulation ratio (*K* = [NCO]/[OH]) were employed.

**Figure 6:**
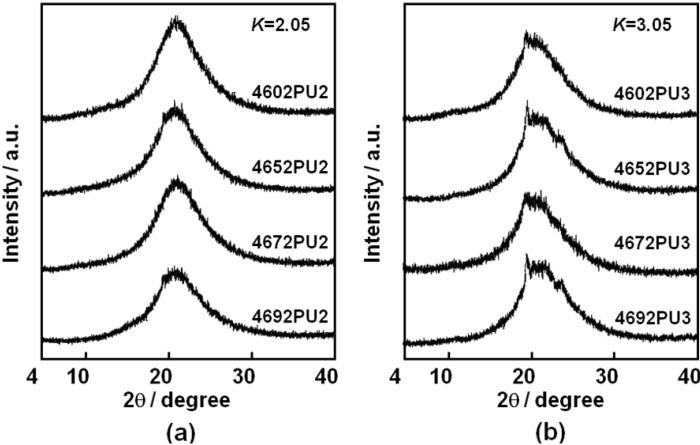
WAXD patterns for PC-glycol-based PUEs. (**a**) *K* = 2.05 and (**b**) *K* = 3.05.

The crystalline state of the PUEs was investigated using WAXD measurement. Figure 6 shows the WAXD profiles of the PC-glycol-based PUEs with (a) *K* = 2.05 and (b) *K* = 3.05. For the PUEs with *K* = 2.05, an amorphous halo was observed. In contrast, WAXD profiles of the PUEs with *K* = 3.05 exhibited crystalline peaks as well as an amorphous halo. WAXD pattern of the hard segment model (-(MDI-BD)_n_-) shows diffraction peaks around 10° and 20° [25,26,27]. Since hard segment content increased with an increase in *K*, it seems that these crystalline peaks in Figure 6(b) are from crystallized hard segment domains. No clear dependence of the C_4_ composition ratio on crystallized hard segment domains was observed for the PUEs.

**Figure 7:**
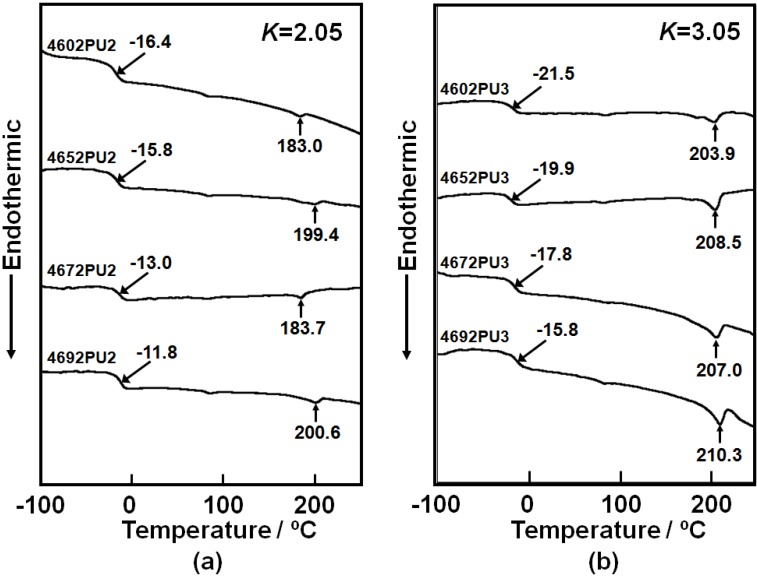
DSC thermograms for PC-glycol-based PUEs. (**a**) *K* = 2.05 and (**b**) *K* = 3.05.

To evaluate the thermal property of the PC-glycol-based PUEs, DSC measurements were carried out. Figure 7 shows the DSC thermograms of the PC-glycol based PUEs with (a) *K* = 2.05 and (b) 3.05. The *T*_g_s of the soft segment were observed from −22 to −10 °C for all PUEs and *T*_g_s of the PUEs with *K* = 3.05 showed lower temperature compared to those with *K* = 2.05. Also, *T*_g_ increased with increasing C_4_ composition ratio for both PUEs. Melting points of the soft segment were not observed for all PUEs, however, those of the hard segment domains were at around 200 °C. Tiny endothermic peaks were observed at around 80 °C. This might be attributed to the melting of short-range hard segment domains or microphase separation transition. It is indispensable for the discussion of the microphase-separated structure to obtain Δ*T*_g_, which is the subtraction between *T*_g_ of the soft segment in the PUEs and *T*_g_ of original polymer glycol. Δ*T*_g_ of the PUEs with *K* = 3.05 exhibited smaller magnitude than for *K* = 2.05. Also, Δ*T*_g_ decreased with an increasing C_4_ composition ratio. The melting point of the hard segment domains of the PUEs with *K* = 3.05 was higher than for *K* = 2.05 and increased with increasing C_4_ composition ratio. Furthermore, the heat of fusion of the melting of the hard segment domains in Figure 7(a) are quite small. Therefore, it seems reasonable to conclude that the microphase separation of the PC-glycol-based PUEs became stronger with increases in *K* and C_4_ composition ratio. This behavior may arise from the lower chain mobility of PC-glycol with high C_4_ composition ratio.

**Figure 8:**
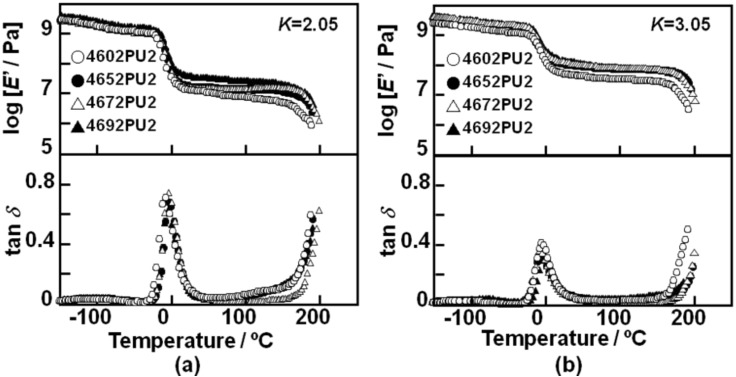
Temperature dependence of storage modulus, *E*’, and loss tangent, tan δ, for PC-glycol-based PUEs. (**a**) *K* = 2.05 and (**b**) *K* = 3.05.

Figure 8 shows the temperature dependence of storage modulus, *E*’, and loss tangent, tan *δ*, for the PC-glycol-based PUEs with (a) *K* = 2.05 and (b) *K* = 3.05. *E*’ and tan *δ* showed typical curves as observed for general thermoplastic polyurethane elastomers. The values of *E*’ in the rubbery plateau region of the PUEs with *K* = 3.05 were larger than for *K* = 2.05, as expected. The order of *E*’ of the rubbery plateau region was 4602 PU < 4652 PU ≃ 4672 PU < 4692 PU for both PUEs with *K* = 2.05 and 3.05. These results correspond well to Young’s modulus and hardness. Peak intensity of tan *δ* of the PUEs with *K* = 2.05 was much larger than for *K* = 3.05. Also, the onset temperature of the tan *δ* peak was observed at around −20 °C, which corresponds well to the *T*_g_ obtained by DSC. Starting temperatures of terminal flow of the 4672 and 4692 PUEs for both *K* = 2.05 and 3.05 were slightly higher than the others. It can be said that introduction of the C_4_ unit improves the heat resistance on account of the increased content of carbonate groups.

**Figure 9:**
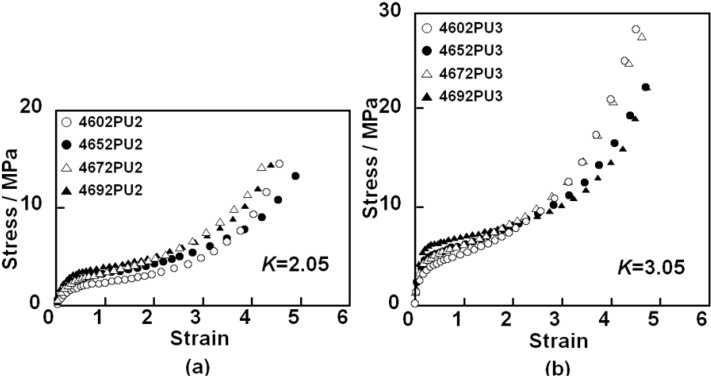
Stress-strain curves for PC-glycol-based PUEs. (**a**) *K* = 2.05 and (**b**) *K* = 3.05.

Mechanical properties of the PC-glycol-based PUEs were evaluated using tensile testing. Figure 9 shows the stress-strain (*ε*) curves for the PC-glycol-based PUEs with (a) *K* = 2.05 and (b) *K* = 3.05. Young’s modulus of these PUEs increased with increases in *K* and C_4_ composition ratio. This is due to increases in hard segment content, the degree of microphase separation and stiffness of polymer glycol with increases in *K* and C_4_ composition ratio. Tensile strength and strain at break showed similar magnitude for the PUEs with each *K*. Steep increases in stress, which is related to the elongation-induced crystallization of the soft segment component, were observed above *ε* = 3 for all PUEs. The slope in this region seems to be correlated to the degree of orientation and/or crystallization of the soft segment chain in the PUEs. 4602 PUs and 4692 PUs exhibited a larger slope compared with 4652 PUs and 4672 PUs for both *K* = 2.05 and 3.05. In other words, the slope of these PUEs decreased, and then increased with increasing C_4_ composition ratio. Elongation-induced crystallization is closely related to the regularity of packing of the glycol soft segment; that is, the symmetry of chemical structure is quite important. The structural irregularities of 4652 and 4672 are much bigger than those of 4602 and 4692. This would be the reason that the slope showed a minimum at around 50% of C_4_ composition ratio.

**Figure 10:**
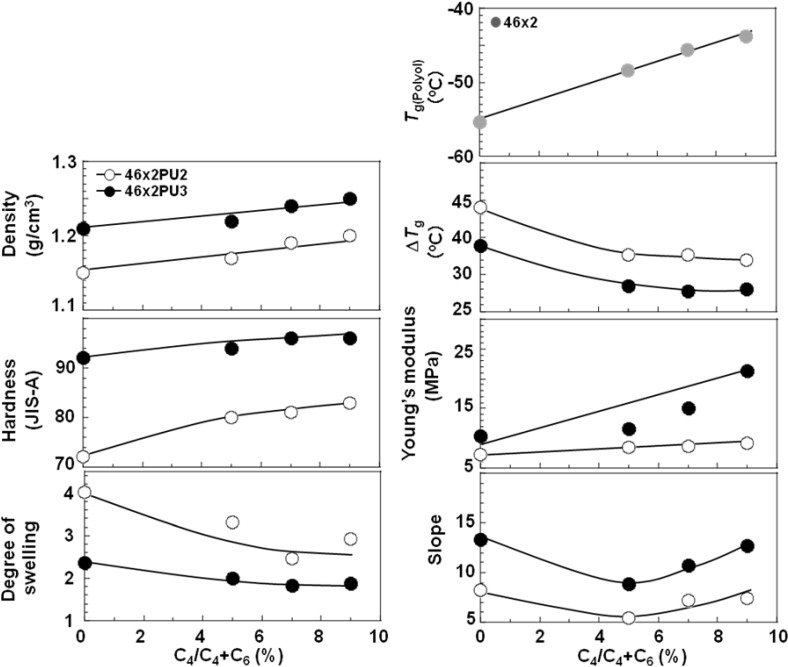
C_4_ composition ratio dependence of various properties for random PC-glycol-based PUEs.

To discuss the C_4_ composition ratio dependence of polycabonate based PUEs, all results are summarized in Figure 10. Figure 10 shows the C_4_ composition ratio dependence of the density, hardness, degree of swelling, *T*_g_, Δ*T*_g_, Young’s modulus and slope around high strain region in the stress-strain curve for all the PC-glycol-based PUEs. Density, hardness and Young’s modulus increased, in contrast, degree of swelling and Δ*T*_g_ decreased with increasing C_4_ composition ratio. On the contrary, the values except Δ*T*_g_ increased with increasing *K*. As stated above, the microphase separation of the PC-glycol-based PUEs became stronger with increases in *K* and C_4_ composition ratio. The trends of hardness and Young’s modulus correspond well to this change in the degree of microphase separation.

Casetta *et al*. reported the effect of side methyl groups of the PC-glycols on the mechanical properties of the PUEs [20]. The *T*_g_s of the randomly copolymerized PC-glycols in this study were lower than for the PC-glycols with methyl side groups. On the contrary, the change in the mechanical properties, for instance, Young’s modulus, tensile strength and strain at break exhibited similar values. Therefore, it seems reasonable to conclude that the introduction of different methylene chains between carbonate groups by random copolymerization is a quite effective method to improve the mechanical properties of the PUEs, similarly to introduction of side methyl groups.

## 4. Conclusions

We prepared PUEs, in which polyether glycols with methyl and dimethyl groups and PC-glycols copolymerized with tetramethylene and hexamethylene groups are incorporated as a soft segment. For the polyether glycol-based PUEs with increasing side methyl groups, the degree of phase separation became weaker and properties of the PUEs were made to soften and weaken as shown by tensile testing. The strain-induced crystallization behavior of the PTMG-based PUEs was controlled by changing the side methyl and dimethyl groups content of PTG-L and PTG-X. WAXD measurement revealed that strain-induced crystallization can be suppressed by the incorporation of 20 mol % of side methyl groups. For the PC-glycol-based PUEs, with increasing C_4_ composition ratio, hardness and Young’s modulus of the PUEs increased due to increases in the degree of microphase separation and stiffness of the polymer glycol. Change in slope in the region, in which elongation-induced crystallization occurred during tensile testing showed a minimum at around 50% of the C_4_ composition ratio. This was explained by the irregularity of the methylene unit of PC-glycols. It was revealed that the introduction of different methylene chain units into PC-glycol is quite effective to control the microphase-separated structure and mechanical properties of the PUEs.
